# Fish bone perforation of the small bowel: A case report

**DOI:** 10.1016/j.amsu.2021.102348

**Published:** 2021-04-27

**Authors:** Abdelilah Elbakouri, Karim Yaqine, Mounir Bouali, Khalid Elhattabi, Fatimazahra Bensardi, Abdelaziz Fadil

**Affiliations:** a Department of General Surgery, University Hospital Centre Ibn Rochd, Casablanca, Morocco; b Faculty of Medecine and Pharmacy, Hassan II University, Casablanca, Morocco

**Keywords:** Fish bone, Foreign body, Perforation, Ileum

## Abstract

**Introduction:**

Fish bone is one of the most common accidently ingested foreign bodies. Normally, it is eliminated from the gastrointestinal (GI) system without any symptomatology, only 1% of the cases will develop a perforation of the GI tract requiring surgical intervention.

**Presentation of case:**

A 70-year-old man, presented with a 48h evolving abdominal pain, important abdominal distension, nausea, vomiting, and a last bowel movement reported 2 days ago, The abdomino-pelvic CT-scan objectified a distension of the terminal ileum measured at 30mm, The exploration revealed a sharp foreign body,at the 15 proximal centimeters of the terminal ileum, which penetrated through the wall of the ileum. The foreign body was removed and we noticed that it is a fish bone. The patient recovered well.

**Discussion:**

Clinical manifestations are determined by the location of the perforation and the preoperative diagnosis is always difficult to reach. Computed tomography (CT) scan is the indicated method to identify ingested foreign bodies and surgery is the treatment of choice.

**Conclusion:**

Delay in diagnosis and treatment can be associated with significant morbidity and mortality.

## Highlights

• Perforations of the gastrointestinal (GI) tract due to an ingested fish bone (FB) are rare.
• Fish bone perforation of the GI is rarely diagnosed preoperatively and has a wide spectrum of clinical presentations.
• Appropriate imagery techniques and a complete interrogatory will lead to the correct diagnosis.
• Delay in diagnosis and treatment can be associated with significant morbidity and mortality.

## 1 Introduction

Accidental ingestion of foreign bodies is a frequently encountered event in clinical practice [1]. Perforations of the gastrointestinal (GI) tract due to an ingested fish bone (FB) are rare (occurring in less than 1% of the cases). The clinical manifestations are mainly determined by the location of the perforation, imaging examinations and surgery are often needed for the diagnosis [2]. We report a case of perforation of the ileum due to a fish bone ingestion.

This work has been reported in line with the SCARE criteria [3].

## 2 Case report

**Fig. 1:**
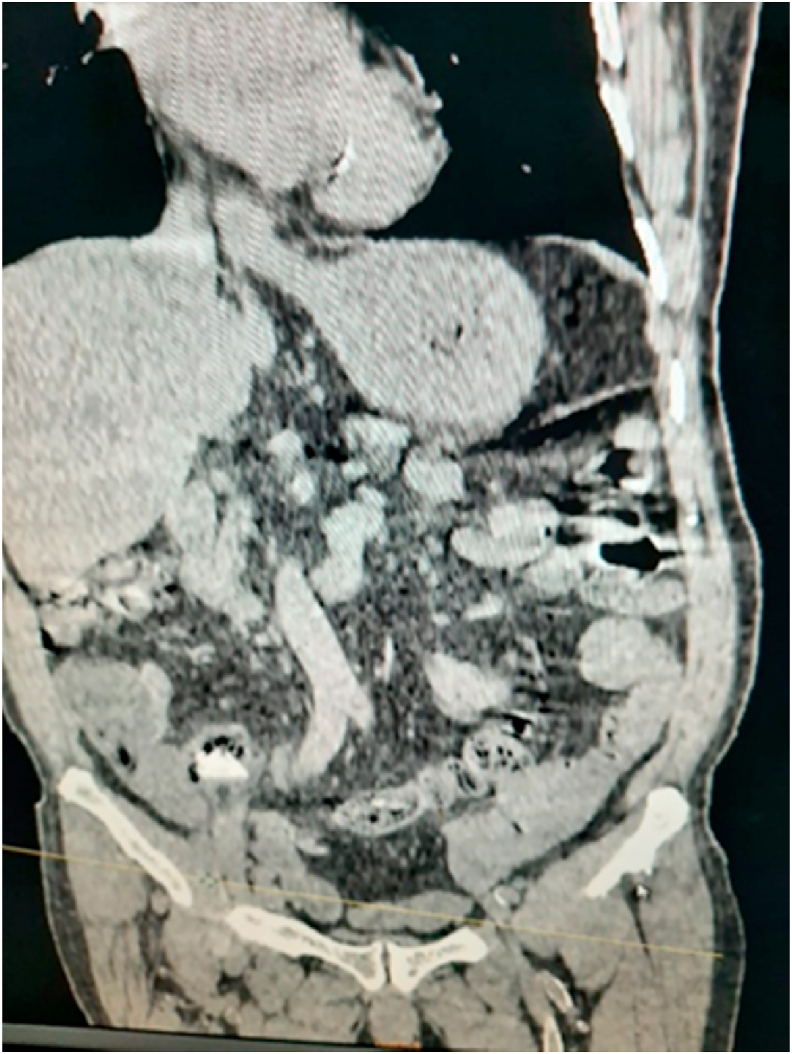
Coronal contrast-enhanced CT-scan of the abdomen showing a hyperdensity indicative of an ingested fish bone.

**Fig. 2:**
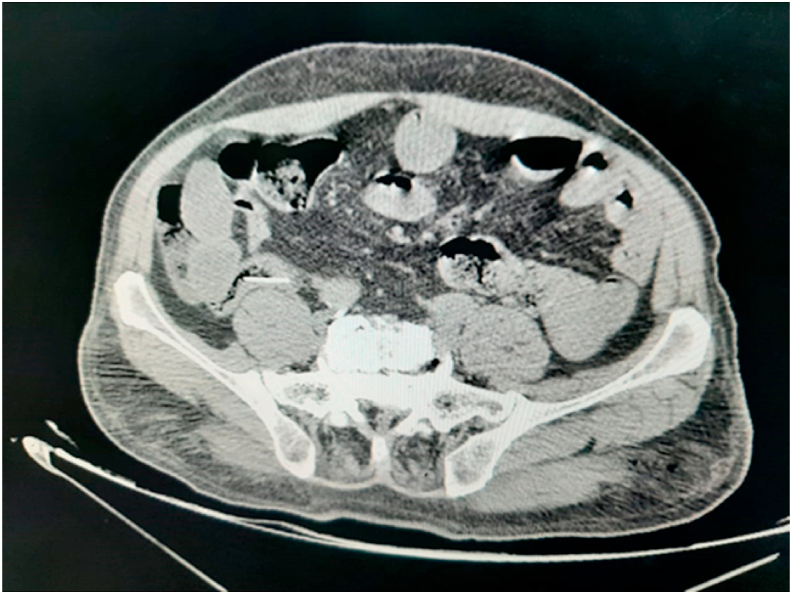
Axial contrast-enhanced CT-scan of the abdomen showing a fish bone.

**Fig. 3:**
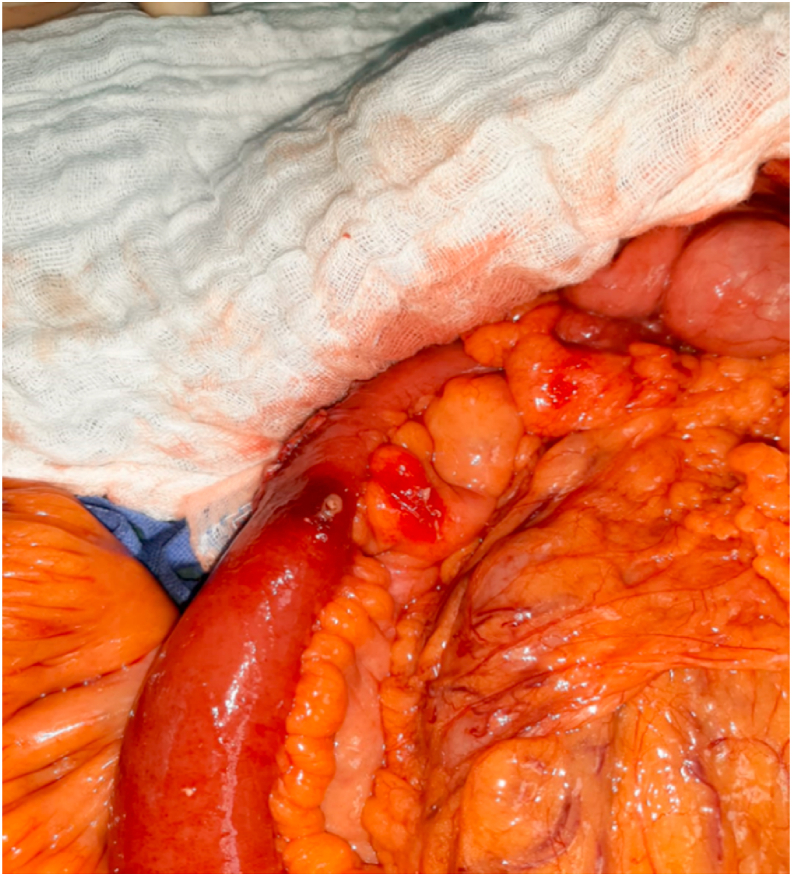
Intraoperative image of the fish bone in the wall of terminal ileum.

**Fig. 4:**
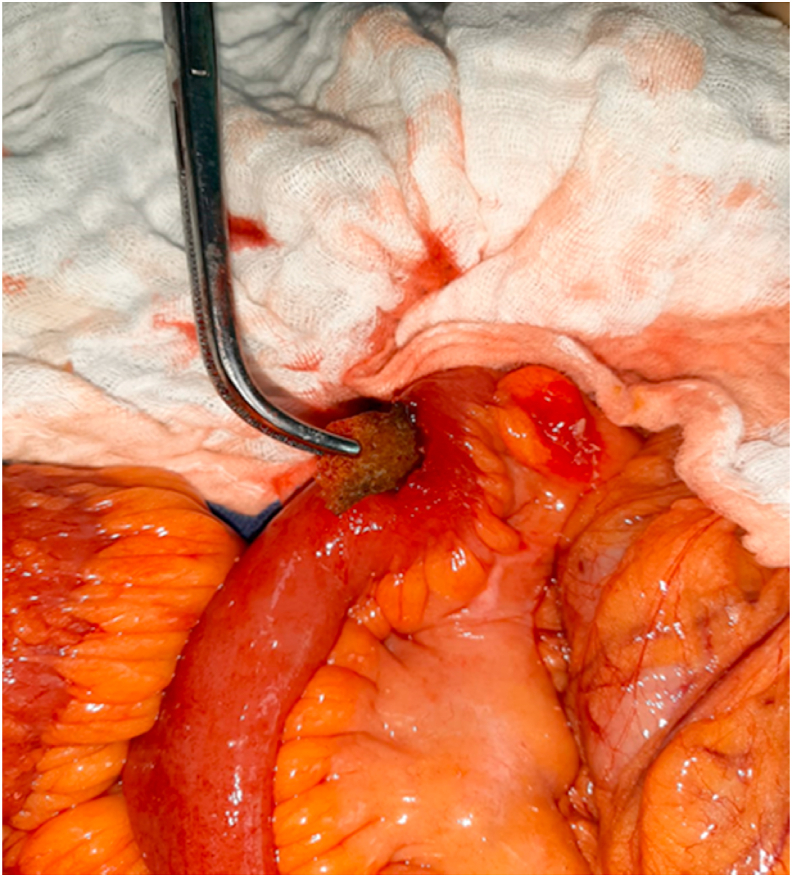
Picture of the fish bone after removal from the lumen of the ileum.

A 70-year-old man, without any particular pathological history, referred by family physician, to emergency with a 48h evolving abdominal pain, important abdominal distension, nausea, vomiting, and a last bowel movement reported 2 days ago. On examination, his vital signs were: temperature: 37.3 °C, pulse rate: 110/minute, respiratory rate: 24/minute and blood pressure: 110/60 mmHg. The abdominal examination revealed an abdominal sensibility at the right illiac fossa, a distension of the abdomen and an empty rectal ampula on rectal examination. The biological checkup showed hyperleukocytosis at 15700/mm3, hemoglobin at 11g/dl, platelets count at 159,000/mm3 and C-reactive protein at 180 mg/liter, the hepatic and renal check-ups were normal. An abdominal X-ray revealed the presence of hydro-aeric levels at the ileum segments without pneumoperitoneum. The abdomino-pelvic CT-scan objectified a distension of the terminal ileum measured at 30mm, upstream of a transitional level in which seats a rectangular and plane foreign body (Fig. 1, Fig. 2). Considering these findings, the patient was diagnosed with intestinal perforation caused by a foreign body. After optimization of his general condition with a Naso-Gastric tube suction and intravenous fluids resuscitation, a decision was taken to proceed with an emergency laparotomy, under general anesthesia with endotracheal intubation. Performed by a 5th year and a 4th year surgical resident. Preoperative prophylactic antibiotics were administered. The exploration revealed a sharp foreign body (fish bone) measuring 3 cm/2cm at the 15 proximal centimeters of the terminal ileum, which penetrated through the wall of the ileum. The foreign body was removed and we noticed that it is a fish bone (Fig. 3, Fig. 4). The margins of the perforation were excised and primary closure was performed. The patient recovered well and was discharged on the 6th postoperative day. On interrogation, the patient acknowledged that he incidentally swallowed the fish bone 5 days ago. The postoperative course was uneventful: improvement of the general condition of the patient.

## 3 Discussion

Foreign body ingestion is a common problem worldwide with an estimated incidence of 120 per million, it is responsible of almost 1500 deaths per year, toddlers are most frequently affected, although rare in conscious and mostly accidentaI, it is fairly a common problem in psychiatric patients [4]. 10–20% of the patients require endoscopic removal and approximately 1% will develop perforation [5]. Fish bone may account for 84% of the foreign bodies ingested accidently, the majority are eliminated from the gastrointestinal system without any symptoms [1].Goh et al. signaled that swallowed fish bones are the most common cause of gastrointestinal perforation due of their sharp tips and long bodies [6]. The most important risk factor for fish bone ingestion is the use of dentures. Other minor risk factors are fast eating, extreme ages (children or elderly), alcohol abuse and mental retardation [7].

The intestine has a remarkable capacity to protect itself against perforation, when the intestinal mucosa is pricked with sharply pointed objects, the bowel wall increases the lumen of the bowel at the point of contact, permitting freer progress to the offending object, the flow of the intestinal contents and the relaxation of the bowel wall tend to make the head lead and the sharp end trail behind [8]. Areas with angulations, a change in direction and transition from a mobile to an immobile segment are considered to be the most vulnerable to perforation by an ingested fish bone. Although fish bone perforation occurs in all segments of the GI tract, the most frequent sites of perforation are the ileum, the ileocecal junction and the rectosigmoid [7]. Injury may occur anywhere from mouth to anus. Also, the perforation may rarely occur at an hernia sac, a Meckel diverticulum or an appendix (8). In our case, an ileal perforation occurred approximately 15 cm from the ileo-caecal valve.

Fish bone perforation of the GI is rarely diagnosed preoperatively and has a wide spectrum of clinical presentations that varies from unseen passage per rectum to severe peritonitis with many clinical manifestations. Depending on the location of the damage, different symptoms can be seen: abdominal pain, vomiting, fever and occasionally melena and bowel obstruction [4]. Different signs have also been reported in the literature including localized abdominal abscess, colorectal, colovesical and enterovesical fistulas, inflammatory mass or omental pseudotumor, bleeding, endocarditis, renal, and ureteral colic. Therefore, the diagnosis is clinically challenging and the first clinical impression is frequently appendicitis or diverticulitis. The stomach, duodenum and the colon tend to have a delayed presentation compared to the perforation of the small bowel [8].

Plain film radiography plays an insignificant role in the detection of fish bones,the sensitivity of detection in the aerodigestive tract is as low as 32%, false negatives are seen in up to 47% of cases [10], sensitivity is influenced by the degree of radio-opacity of the bone depending on the species of the fish. Even when fish bones are sufficiently radio-opaque, large soft tissue masses and fluid can obscure the minimal calcium content of the bone, particularly whith obese patients [8]. The presence of pneumoperitoneum is not reliable as it is not found in many cases, the perforation is usually caused by the impaction and the progressive erosion of the FB through the intestinal wall, allowing it to be covered by fibrin, omentum and adjacent loops of bowel [6]. With recent advances in the image quality of the CT-scans, the ability to identify a fish bone in a lesion is much better. Multi detector CT-scan is the method of choice to evaluate patients with acute abdominal pain and to detect foreign bodies, it may provide detailed examination from all aspects with high resolution and multi-planed reconstruction abilities [9]. Associated CT-scan findings at the site of perforation include: mucosal wall thickening of the bowel, intestinal obstruction, pericolic fat stranding, and at times there may be abscess formation [10]. In some cases, imagery findings can be non specific; however, the finding of a foreign body with extra-luminal pockets of free air or an associated mass in patients with clinical signs of peritonitis, mechanical bowel obstruction or pneumo-peritoneum strongly suggests the diagnosis of foreign body perforation [4]. Potential pitfalls on the CT-scan include the presence of positive bowel contrast, cricoid cartilage calcification, artifacts related to fecal material within the colon and contrast opacified small blood vessels which can mimic a fish bone [1].

The management of an ingested foreign body depends on the patient symptoms and the type and the location of the ingested object [11]. Surgery is the treatment of choice to repair any perforation caused by foreign body, upon development of complications such as abscess, fistula, and ileus. Surgical treatment of small intestine perforations require surgical repair or segmental resection. Depending on the size of the perforation, the degree of contamination, the underlying condition of the bowel and the judgement of the surgeon, an early intervention should be taken to prevent further morbidity and mortality [9].

## 4 Conclusion

Perforation of intestinal structures by fish bone is a challenging diagnosis that should always be recalled in cases of acute abdominal symptoms. Appropriate imagery techniques and a complete interrogatory will lead to the correct diagnosis. Delay in diagnosis and treatment can be associated with significant morbidity and mortality.

## Provenance and peer review

Not commissioned, externally peer reviewed.

## Sources of funding

None.

## Ethical approval

I declare on my honor that the ethical approval has been exempted by my establishment.

## Consent

Written informed consent for publication of their clinical details and/or clinical images was obtained from the patient.

## Author contributions

Abdelilah Elbakouri: writing the paper.

Karim Yaqine: Corresponding author writing the paper.

Mounir Bouali: study concept.

Khalid Elhattabi: correction of the paper.

Fatimazahra Bensardi: correction of the paper.

Abdelaziz Fadil: correction of the paper.

## Trial registry number

Not applicable.

## Guarantor

DOCTEUR YAQINE KARIM.

## Declaration of competing interest

The authors declare having no conflicts of interest for this article.
